# Explaining Away, Augmentation, and the Assumption of Independence

**DOI:** 10.3389/fpsyg.2020.502751

**Published:** 2020-11-03

**Authors:** Nicole Cruz, Ulrike Hahn, Norman Fenton, David Lagnado

**Affiliations:** ^1^Department of Psychological Sciences, Birkbeck, University of London, London, United Kingdom; ^2^School of Electronic Engineering and Computer Science, Queen Mary University of London, London, United Kingdom; ^3^Department of Experimental Psychology, University College London, London, United Kingdom

**Keywords:** intercausal reasoning, explaining away, noisy-or, uncertain evidence, negative evidence

## Abstract

In reasoning about situations in which several causes lead to a common effect, a much studied and yet still not well-understood inference is that of *explaining away.* Assuming that the causes contribute independently to the effect, if we learn that the effect is present, then this increases the probability that one or more of the causes are present. But if we then learn that a particular cause is present, this cause “explains” the presence of the effect, and the probabilities of the other causes decrease again. People tend to show this explaining away effect in their probability judgments, but to a lesser extent than predicted by the causal structure of the situation. We investigated further the conditions under which explaining away is observed. Participants estimated the probability of a cause, given the presence or the absence of another cause, for situations in which the effect was either present or absent, and the evidence about the effect was either certain or uncertain. Responses were compared to predictions obtained using Bayesian network modeling as well as a sensitivity analysis of the size of normative changes in probability under different information conditions. One of the conditions investigated: when there is certainty that the effect is absent, is special because under the assumption of causal independence, the probabilities of the causes remain invariant, that is, there is no normative explaining away or augmentation. This condition is therefore especially diagnostic of people’s reasoning about common-effect structures. The findings suggest that, alongside earlier explanations brought forward in the literature, explaining away may occur less often when the causes are assumed to interact in their contribution to the effect, and when the normative size of the probability change is not large enough to be subjectively meaningful. Further, people struggled when given evidence against negative evidence, resembling a double negation effect.

## Introduction

Imagine you are on a tropical island in which there are three types of mosquito (Reb, Mar, and Murb) that carry a disease, called Ling fever. For each mosquito type, there is a risk of being bitten by an infected mosquito, and a risk of contracting the disease when bitten. One day during a routine health check, it turns out that you have Ling fever, prompting you to increase your degree of belief that you were bitten by an infected mosquito. Further tests show that you were bitten by an infected mosquito of the Reb type. How does this additional information affect your degree of belief that you were bitten by an infected mosquito of the Mar type? In this situation, the presence of a bite from Reb “explains away” the finding of Ling fever, suggesting one can reduce one’s degree of belief in a bite from Mar (Rehder and Waldmann, 2017).

Now imagine the further test showed instead that you were *not* bitten by an infected mosquito of the Reb type. How does this additional piece of information affect your degree of belief that you were bitten by an infected mosquito of the Mar type? In the absence of a bite from Reb, the finding of Ling fever is still in need of an explanation, suggesting one can “augment” one’s degree of belief in a bite from Mar.

The above reasoning is called intercausal because it involves inferring the likelihood that one cause is present or absent, based on knowledge about one or more further causes. People have been found to show explaining away and augmenting in intercausal reasoning tasks, though not always reliably. In particular the size of the effects has sometimes been smaller than predicted (Morris and Larrick, 1995; Ali et al., 2011; Rottman and Hastie, 2014; Liefgreen et al., 2018; Tešić et al., 2020). The present paper aims to investigate further the conditions under which these inferences are drawn. It explores to what extent people change their intuitions about augmenting and explaining away as a function of (a) whether the evidence about the effect is positive or negative, and (b) whether this evidence is certain or uncertain. But before going into more detail about these two factors, let us turn briefly to the general framework within which changes in people’s degrees of belief like those of explaining away and augmentation can be represented.

Changes in degrees of belief over time as new information about a situation becomes available can be modeled in a Bayesian network (BN) (Pearl, 1988, 2000). In a BN, the relevant events are represented as variables and arrows represent (non)independence relations connecting the variables, forming a directed acyclic graph (DAG). Associated with each variable is a conditional probability table (CPT), which specifies the probability of each value that the variable can take, as a function of each of the possible values of the variables on which it directly depends (i.e., is linked to by arrows). In this way, BNs allow the graphical representation and variation of complex probabilistic relations between events, making transparent which variables are positively or negatively related to one another, and which are independent, and supporting the computation of dynamic changes to beliefs as evidence comes in. This probabilistic, Bayesian approach to causal reasoning provides an alternative to earlier approaches based on classical logic (Fernbach and Erb, 2013; Oaksford and Chater, 2017; Over, 2017), possible worlds semantics (Lewis, 1973; Stalnaker, 1981; Briggs, 2012), and theories of associative learning (Waldmann and Holyoak, 1992; Sloman and Lagnado, 2005, 2015; Rehder, 2014).

**FIGURE 1:**
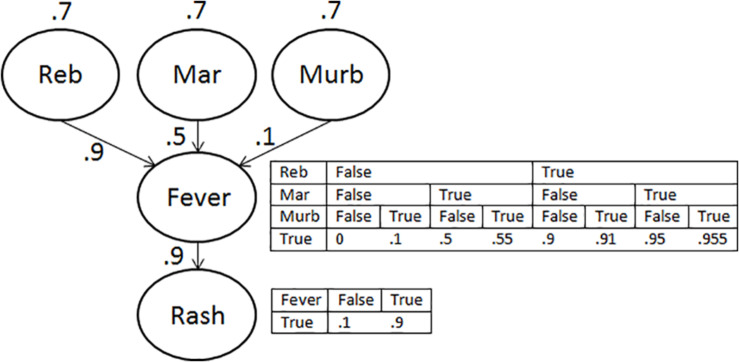
Causal structure of the scenario. The upper conditional probability table (CPT) displays the probability of fever given the presence or absence of each of the three causes. The lower CPT shows the probability of a rash given the presence or absence of fever. These CPTs follow from the priors and causal power values shown in the graph, together with a leakage parameter of 0 for Fever, and of 0.1 for Rash.

A BN for our mosquito example has three (marginally independent) causes (a bite of an infected mosquito of type Reb, Mar, or Murb), and one common effect (Ling fever). Such a structure is shown in Figure 1. The CPT for the effect would then contain the probability of Ling fever for each combination of the truth or falsity of each of the three causes, yielding eight distinct entries like those shown. People may not have clear intuitions about the probability of each of the eight entries, but fewer parameters need to be specified if one can draw on a more general function specifying how the impact of the causes combines to bring about (or prevent) the effect (c.f. Fenton et al., 2007).

A typical function for common-effect structures like that of the mosquito example is the *noisy-or*. The noisy-or specifies the probability of the effect given a disjunction of independent causes. It is a generalization of the Boolean OR to reasoning from uncertain premises. The basic idea is that the probability of a disjunction is equal to 1 minus the probability of the negation of the disjunction, so that P(effect| *A or B*) = 1 – P(effect| *not-A & not-B*). Formally, let *x*_i_ = *x_1_,..,x_*n*_* be *n* variables representing the causes of an effect *y*. Let *v*_i_ be a weight factor for each cause, specifying the conditional probability of the effect given cause *i* in the absence of the other causes (i.e., the causal power of cause *i*, Cheng, 1997). Finally, let *λ* be a *leakage parameter* specifying the probability that the effect occurs when all the causes included in the model are absent. The leakage parameter is like a residual category covering the impact of any causes that have not been explicitly specified. Then the probability of the effect is given by:

$$\left(y=1|x_{1},\ldots ,x_{n}\right)=1-\left(1-\lambda \right)\prod_{i=1}^{n}\left(1-v_{i}\right)$$

where (*y* = 1|*x*_1_,…,*x*_*n*_) stands for the probability of the effect under the noisy-or, $\prod_{i=1}^{n}\left(1-v_{i}\right)$ calculates the probability of the effect given that all causes are absent, (1-λ) specifies that also all not explicitly represented causes are absent, and finally, 1− takes the complement to arrive at the probability of the effect given that one or more causes are present, that is the probability of the noisy-or. When all weight parameters *v*_i_ are 1 and the leakage parameter *λ* is 0, then the noisy-or reduces to the Boolean OR.

The definition of the noisy-or function implies that the causes are marginally independent (such that in the absence of further information, the presence or absence of one cause does not affect the probability that other causes are present or absent) and it implies that the causes contribute independently to the effect. This means that the causal power *v*_i_ of one cause does not change with the presence or absence of other causes. In the mosquito example, one would say that the probability of contracting Ling fever from a bite of Mar remains the same whether or not we have also been bitten by Reb.

The noisy-or is the most widely used function for specifying the CPT entries in common effect structures, and experimental materials in causal reasoning research are often constructed with the aim of instantiating its independence assumptions. When these assumptions are met for a given situation or scenario, then it is possible to use the noisy-or to define the normative probability of the effect under different values of the causes. Sometimes the independence assumptions of the noisy-or have also been proposed to be descriptive of people’s reasoning with common effect structures in general (Griffiths and Tenenbaum, 2009; Holyoak and Cheng, 2011), and findings of responses deviating from these assumptions have been explained as arising from people adding further information to the scenario that changes the original common effect structure into a different one (Mayrhofer et al., 2010; Rehder, 2014). In line with the default use of the noisy-or to model common effect structures, there is evidence that people find independent, additive relations between variables easier to process than interactive relations (Juslin et al., 2009; Cruz and Oberauer, 2014; Rehder and Waldmann, 2017). However, the default use of the noisy-or has also recently been criticized, partly because of concerns that it might not always be a realistic representation of causal relations in the world (Fenton et al., 2019; Noguchi et al., 2019). There can be cases in which the causes do not act independently but instead enhance or inhibit each other’s contribution to the effect, and people may sometimes take account of such departures from independence in their reasoning.

This paper assesses predictions derived from the independence assumptions of the noisy-or under different conditions, and compares them to those expected under the assumption of enhancement. Inhibitory causal interaction was not considered here, but would also be worth investigating further. In the mosquito example, independent contributions of the causes to the effect can be thought of as establishing a linear relation between the number of bites from infected mosquitos and the probability of Ling fever. Causes that enhance each other’s contribution to the effect could be thought of as establishing an exponential relation between number of bites and probability of Ling fever, as if once arriving in the hosts’ body, the Ling bacteria coordinated their behavior to make the disease break out.

Below we discuss the predictions for independence in relation to the four conditions that result from crossing (a) whether the evidence for the effect is positive or negative, and (b) whether this evidence is certain or uncertain – and discuss how these predictions would change under the assumption of enhancement.

### Condition 1: Certain Positive Evidence

Suppose we learn that the effect is present (we have Ling fever), and so increase our degree of belief in the causes (a bite from an infected mosquito of any type). If we then go on to learn that a particular cause A (e.g., a bite from Reb) is present, this “explains away” the presence of the effect. Under the noisy-or it is then normative to *decrease* again our degree of belief in the other causes (Mar and Murb). In the limit, when P(effect|cause A) = 1, cause A “explains” the presence of the effect entirely, and the probability of the other causes decreases all the way back to its baseline – the value it had before receiving the information that the effect was present. Suppose we instead go on to learn that cause A is absent. Then we are still in need of an explanation for the effect, and it is normative to augment, or increase, the probability of B. Hence under the independence assumption of the noisy-or, Condition 1 leads to the prediction of explaining away of a cause B when another cause A is present, and it leads to augmentation of a cause B when another cause A is absent.

How can the causes affect one another in this way under the noisy-or, even though they are marginally independent? When causes are *marginally independent*, then in the absence of further information, knowing that one cause is present or absent does not change the probability that another cause is present or absent. But once we learn that the effect has occurred, the causes become c*onditionally dependent* on the presence of the effect. The effect establishes an indirect connection between the causes, making information about the presence or absence of one cause informative about the presence or absence of another.

### Condition 2: Uncertain Positive Evidence

Suppose we do not know for sure that the effect (Ling fever) is present, but only have some uncertain indirect evidence for the effect because a consequence of the effect (e.g., a rash) is present. Then this evidence again renders the causes dependent, and it is normative under the noisy-or to show the same pattern of explaining away and augmentation as in Condition 1. The impact of uncertainty in Condition 2 is merely to decrease the size of the normative changes in probability.

### Condition 3: Certain Negative Evidence

Suppose we come to know for certain that the effect is absent (we do not have Ling fever). Then it is normative to decrease our degree of belief in the causes. However, under the noisy-or the causes remain independent in this case. Additional information showing that one cause is present or absent does not undo our certainty about the absence of the effect, and so will not alter our degree of belief in the presence or absence of the other causes. Hence there is normatively no explaining away or augmentation under the noisy-or in Condition 3. It was precisely this concern about noisy-or that was addressed in Fenton et al. (2019) and Noguchi et al. (2019).

### Condition 4: Uncertain Negative Evidence

Finally, suppose the effect (Ling fever) is not known for certain to be absent, but there is only some uncertain indirect evidence for this because its consequence (rash) is absent. Then the probability of the causes decreases, albeit by a smaller amount than when knowing the effect to be absent with certainty. However, because of the lingering uncertainty about whether the effect is really absent, the causes become dependent under the noisy-or. Additional information showing that one of the causes is present or absent can reduce or increase our uncertainty about the absence of the effect, and as a result, becomes informative about the probability that another cause is present or absent. Specifically, the presence of a particular cause A increases the probability of the effect, partly canceling out the reduction in the probability of the effect brought about by the absence of its consequence. As a result, the probability of an alternative cause B increases. Conversely, when A is absent, this decreases the probability of the effect, adding to the reduction in the probability of the effect brought about by the absence of its consequence. As a result, the probability of B decreases further. This pattern of probability changes goes in the opposite direction to that of explaining away and augmentation of Conditions 1 and 2.

What would follow for these four conditions if the causes did not contribute independently to the effect, but instead enhanced each other’s impact? The previously described mechanisms of probability change would still be in place, but they would be overlaid by additional changes in probabilities resulting from the positive correlation between the causes. Which changes in probability prevail will depend on the relative weight of the prior probabilities and effectiveness of the causes on the one hand, and the correlation between the causes on the other.

When a positive correlation between causes is small relative to their prior probabilities and effectiveness to bring about the effect, then the direction of probability changes will be the same as for the noisy-or, although augmentation effects will be larger and explaining away effects smaller. When the prior probabilities and effectiveness of the causes are small relative to the correlation between the causes, then the impact of the correlation can override the effects predicted under independence, potentially flipping the direction of probability changes. For example, for the structure of Figure 1, if we know we have Ling fever and were bitten by a Reb type mosquito, then this decreases the chances that we were also bitten by a Mar type mosquito under independence. However, in a situation in which Reb and Mar very rarely bite, but when they do, they almost always bite together, then learning we were bitten by Reb might instead increase the chances that we were also bitten by Mar.

The current study did not explicitly manipulate the correlation between causes, and instead went a step back to first assess whether people’s responses followed a pattern consistent with presence or absence of a correlation when this question was left open. However, the priors and effectiveness values used in this study, together with the absence of information about a potential correlation between causes, suggest it is unlikely that participants will assume a correlation between causes high enough to override the impact of priors and effectiveness information. Therefore, under the assumption of enhancement we expect explaining away to be lower in Conditions 1 and 2 than it would be under independence, but we do not expect response patterns in these conditions to flip qualitatively into augmentation. Similarly, in Condition 4 we expect the assumption of enhancement to increase the size of augmentation effects and decrease the size of explaining away effects relative to their values under independence, but we do not expect a qualitative flip from explaining away to augmentation or vice versa.

In contrast, Condition 3 does involve a qualitative difference in the predicted response patterns under assumptions of independence and of enhancement. When the effect is known to be absent with certainty, there is no explaining away or augmentation under independence. In contrast, under enhancement we expect a similar pattern of explaining away and augmentation to that predicted under the noisy-or for conditions 1 and 2, albeit again attenuated for explaining away and accentuated for augmentation. Condition 3 therefore provides a unique opportunity to differentiate whether people are interpreting causes as independent or correlated.

The above predictions are based on general principles of probability theory in a Bayesian network framework, as outlined for example in Wellman and Henrion (1993) or Morris and Larrick (1995), along with Bayesian network modeling to obtain more precise quantitative predictions for different model parameterizations (see discussion section). Table 1 summarizes the predictions under the noisy-or for the four conditions described above.

**TABLE 1:** Predictions under the noisy-or for the direction of probability change of a cause B after learning that another cause A is present or absent, given four different types of evidence for the effect.

	**A present**	**A absent**
(1) Certain positive evidence	B decreases	B increases
(2) Uncertain positive evidence	B decreases slightly	B increases slightly
(3) Certain negative evidence	B remains invariant	B remains invariant
(4) Uncertain negative evidence	B increases slightly	B decreases slightly

*See the general discussion for quantitative predictions for different parameterizations.*

In contrast to the extensive empirical work using noisy-or structures with positive certain evidence, there has been very little research about situations involving negative evidence, uncertain evidence, or common-effect structures that do not conform to the independence assumption of the noisy-or but instead have causes that are correlated or interact (Wellman and Henrion, 1993; Morris and Larrick, 1995; Rehder, 2014; c.f. Rottman and Hastie, 2014). For example, in one group of experiments (Rehder, 2014) participants were asked to assume that two causes contributed independently to a common effect, using relatively abstract scenarios with no information about the marginal probability of each cause. Participants were asked to compare the probability of a cause in two situations that differed in terms of whether the other cause and the effect were present, absent, or their state was unknown. When the effect was absent, participants tended to judge a given cause as equally likely regardless of the value of the other cause, as predicted by the noisy-or (case 3 above). But they also tended to judge the cause as equally likely in situations in which one would have predicted explaining away to occur. The authors explained this pattern, which is not predicted by any theory, as an aggregate of a group of participants following the predictions of the noisy-or, and another group establishing not causal but associative links between the variables involved. Associative links differ from causal links by being bidirectional rather than unidirectional. However, further research is needed to explore alternative interpretations of these findings (see Tešić et al., 2020). Earlier studies on people’s sensitivity to the impact of interactions between causes when these are made explicit in the instructions (Morris and Larrick, 1995) suggest that people’s intuitions do capture the direction of the changes in probability that follow from such interactions. But the extent of such intuitions, and the contexts in which they arise, are as yet underexplored.

This paper presents an experiment intended to be a first step in assessing people’s intuitions for the four conditions outlined above. To our knowledge, this is the first time that predictions under causal independence and under causal enhancement are compared directly in a single experiment, with respect to both explaining away and augmentation, and for both positive and negative evidence about the effect. The comparison also takes into account the differential impact of whether this evidence is certain or uncertain. We compared these conditions using the above mosquito scenario, which given its fictional contents was considered relatively open with respect to how the causes integrate their impact to bring about the effect. Overall, we aimed to assess which of the two integration functions accounts better for people’s responses when the nature of the function is not prespecified, while taking into account that people may be uncertain about the information given even when instructed to assume it to be true or false with certainty (Evans and Over, 2004; Oaksford and Chater, 2007, 2013; Pfeifer and Kleiter, 2009; Over and Cruz, 2018).

## Materials and Methods

### Participants

Fifty residents of English speaking countries completed the online experiment via the platform Prolific Academic, providing informed consent for participation. After excluding the data of participants with speeded trial responses, failed attention checks, and modest reported English language skills, the final sample consisted of 37 participants. They had a median age of 39 (range 22–65), and had a diverse formal educational background.

### Materials and Design

At the start of the experiment and then again at the top of each trial, participants were shown information about a fictional archipelago in which three types of Mosquito (the Reb, Mar, and Murb mosquito) could transmit a disease known as Ling fever. The information about the mosquitos and the disease reflected the causal structure in Figure 1. Participants were informed that the prior probability of being bitten by an infected mosquito was 70% for each type, but that the mosquito types differed in the effectiveness with which they transmitted the disease when they bit their hosts. In the absence of bites from the other two mosquito types, the bite of an infected Reb mosquito led to Ling fever 90% of the time; the bite of an infected Mar mosquito led to Ling fever 50% of the time; and the bite of an infected Murb mosquito led to Ling fever 10% of the time. Ling fever could not be contracted through any other cause (i.e., the leakage parameter for the effect was 0). A person with Ling fever had a 90% chance of showing a purple rash. A purple rash due to other causes occurred only 10% of the time on the archipelago. The scenario made no statement about the presence or absence of any relation between causes. The above combination of parameters was chosen on the basis of a prior exploration of the parameter space in which the Bayesian network structure of Figure 1 was queried using parameter values across the probability range, with the aim of maximizing the size of normative probability changes across conditions. The sizes of normative changes nonetheless never exceeded 20% and were sometimes smaller than 10%. We discuss the implications of this limitation further below.

**FIGURE 2:**
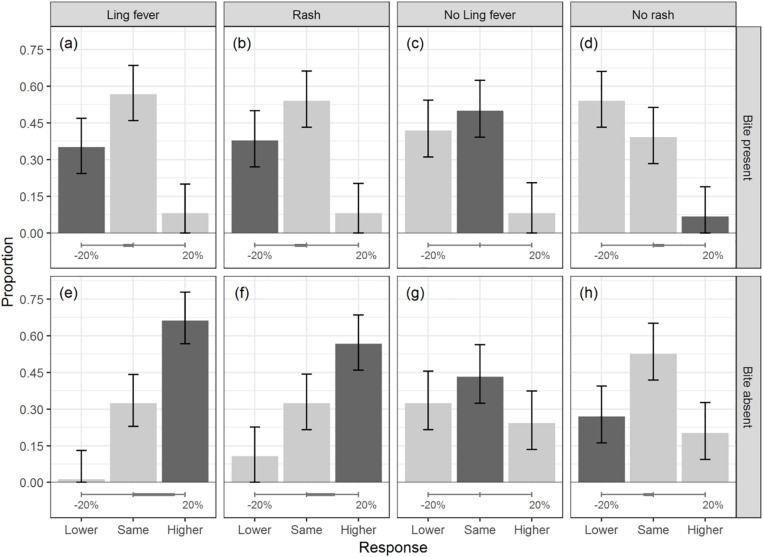
Proportion of times each of the three response options (*lower, same, higher*) was chosen in each of the eight experimental conditions. The rows show the data separately for when the cause was present **(upper)** vs. absent **(lower)**. The columns show the data separately for the conditions in which the effect was present (Ling fever), its consequence was present (rash), the effect was absent (no Ling fever), or the consequence of the effect was absent (no rash). The dark gray bar in each panel represents the normative response under independence. The horizontal scale at the bottom of each panel shows the size of the normative change under independence. Error bars show 95% CIs.

The design crossed two within participant variables: (1) initial information about the effect, that is whether the effect was present (Ling fever), the effect was absent (No Ling fever), the consequence of effect was present (Rash), or the consequence of effect was absent (No rash); and (2) additional information about one of the causes (bite present vs. bite absent). Crossing these two variables resulted in eight conditions, reflected in the eight panels of Figure 2 below.

For each of the eight conditions there were two trials, yielding 16 trials in total. On one of the trials, participants were informed that a protagonist was or was not bitten by an infected Reb mosquito, and were asked what impact this information had on the probability that the protagonist was bitten by an infected Mar mosquito. On the other trial, participants were informed that a protagonist was or was not bitten by an infected Mar mosquito, and were asked what impact this information had on the probability of them being bitten by an infected Reb mosquito. The difference between these two trials comes from the difference in effectiveness of disease transmission between mosquito types. As mentioned above, a bite from an infected Reb mosquito causes Ling fever 90% of the time, whereas a bite from an infected Mar mosquito causes Ling fever only 50% of the time. Hence, on one trial information about the presence/absence of a cause with high effectiveness is used to draw an inference about the presence/absence of a cause with medium effectiveness, and vice versa on the other trial. The Murb mosquito did not feature in the questions asked to participants because with only 10% effectiveness, this cause was associated with only very small normative changes in probability across conditions. The role of the differential effectiveness of the causes can be related to research on the reliability of testimony (Hahn et al., 2013). However, this variable goes beyond the scope of the questions addressed in this paper and its results are not discussed further here. In the context of this paper, cause effectiveness is merely a methodological variable whose inclusion makes it possible to generalize the results on the questions of interest to more than one effectiveness value. The results presented were thus averaged across the two trails for each of the eight cells of the design.

On each trial, participants were given initial information about the effect, and additional information about one of the causes. The task was to judge whether after receiving the additional information, the probability of a second cause was higher, lower, or the same compared to before receiving the additional information. The order of trials was randomized for each participant. A screenshot of a sample trial from Condition 1 (certain positive evidence) is shown below. Each trial referred to a different island and protagonist.

Initial information for the island of Eik:

• The risk of being bitten by an infected mosquito is the same for the three mosquito types. Within a given month, a random person from the island has a 70% chance of being bitten by an infected Reb mosquito, a 70% chance of being bitten by an infected Mar mosquito, and a 70% chance of being bitten by an infected Murb mosquito.
• But the species differ in the effectiveness with which they transmit the disease when they bite their hosts.
• The Reb mosquito transmits the disease 90% of the time that it bites; the Mar mosquito 50% of the time, and the Murb mosquito 10% of the time.
• A person that has the disease has a 90% chance of showing a characteristic purple rash. The chances that a person from the island would show such a rash for other reasons is only 10%.

**Michele from the island of Eik is known to have Ling fever. A further test showed that Michele was bitten by an infected Mar mosquito. Does this additional information change the chances that Michele was bitten by an infected Reb mosquito? If so, then in which way?**

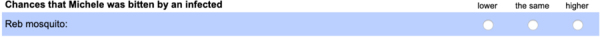

We asked participants to provide qualitative judgments of probability changes rather than to make repeated quantitative probability judgments under different information conditions, because we wanted to make the task less dependent on numeracy as well as on working memory limitations that could have an impact when comparing responses across trials. However, we did ask for percentage probability judgments during eight practice trials aimed at allowing participants to form an impression of the relevant causal structure and the relations between the probabilities of its elements. Two of the practice trials asked for P(cause A & cause B) and P(cause A or cause B). These probabilities allowed us to obtain an indirect impression of whether participants perceived the causes to be initially independent, that is, whether P(*A & B*) = P(*A*)P(*B*), and P(*A or B*) = P(*A*) + P(*B*) − P(*A & B*). We computed probabilistic coherence, that is, conformance with the axioms of probability theory, of people’s responses to these two questions with and without the assumption of cause independence. This is an indirect measure because people’s responses could be incoherent for many reasons (Tversky and Kahneman, 1983; Bar-Hillel and Neter, 1993). But it provides one source of information on the question, which can then be complemented with further information from this and future experiments.

### Procedure

Participants went through the instructions and eight practice trials, followed by the 16 trials of the main experiment. The information on the causal structure and parameters for the scenario remained visible on each trial. At the end of the experiment, participants provided demographical information and were asked to rate on a percentage scale how difficult they found the task. The median rating of experiment difficulty was 74%. The median duration of the experimental session was 12.23 min.

## Results and Discussion

Coherence for participants’ responses to the two practice trials asking for P(*cause A & cause B*) and P(*cause A or cause B*) was computed by first coding whether a given response was coherent or not – separately under the assumption of independence and without this assumption – and then subtracting the resulting variable for observed coherence from the chance rate of obtaining a coherent response, in order to determine whether responses were coherent more often than expected by chance (Cruz et al., 2015; Evans et al., 2015). The chance rate of a coherent response under independence is constrained to a point value as given by the equalities P(*A & B*) = P(*A*)P(*B*), and P(*A or B*) = P(*A*) + P(*B*) − P(*A & B*). In contrast, the chance rate of a coherent response without making any assumption about the relation between the causes is an interval on the probability range. For the probability of the conjunction of two causes A and B, this interval is [max(0, P(*cause A*) + P(*cause B*) − 1), min(P(*cause A*), P(*cause B*))]. For the probability of the disjuncti on of two causes it is [max(P(*cause A*), P(*cause B*)), min(P(*cause A*) + P(*cause B*), 1)].

The coherence of participants’ responses to the two practice questions was found to be at chance level under the assumption of independence, but above chance when not making any assumption about how the causes might or might not be related. Specifically, assuming independence, responses to the conjunction question were coherent 7% more often than expected by chance (*t*(36) = 1.56, *p* = 00.127, 95% CI [−0.021,0.163]); and responses to the disjunction question were coherent 2% more often than expected by chance (*t*(36) = 0.53, *p* = 0.533, 95% CI [−0.038,0.072]). This outcome did not change when the range of coherent responses was increased by + −5%, and the chance rate increased accordingly, to account for the possibility that people are sensitive to the relevant coherence constraints but have degrees of belief that are coarser than point probabilities. In contrast, without assuming any specific relation between the causes, responses to the conjunction question were coherent 54% more often than expected by chance (*t*(36) = 8.75, *p* < 0.001, 95% CI [0.413,0.662]); and responses to the disjunction question were coherent 65% more often than expected by chance (*t*(36) = 17.14, *p* < 0.001, 95% CI [0.570,0.722]).

This finding does not in itself suggest that people are not assuming the causes to be independent, or that they are making no assumption about the relation between causes. But it provides an initial indication that people’s probability judgments in experiments may sometimes become more understandable when moving beyond the presupposition of independence.

The pattern of responses in the main experiment is displayed in Figure 2. The x axis shows the three response options, and the height of the bars represents the proportion of times a response was chosen within each of the eight conditions. The darker bar in each panel shows the predicted response under independence. Each column of the figure corresponds to one of the four conditions in Table 1: effect present (Ling fever), consequence of effect present (rash), effect absent (no Ling fever), and consequence of effect absent (no rash). The first row represents the conditions in which an alternative cause was present, and the second row the conditions in which an alternative cause was absent. The horizontal scale at the bottom of each panel represents the size of the normative change under independence. An initial look at the figure tells us that the normative response under independence was the numerically most frequent in four of the eight experimental conditions (panels c, e, f, and g). The normative response under the assumption of a modest positive correlation between causes, whose impact is not stronger than that of the causes’ priors and effectiveness values, was numerically most frequent in three of the eight conditions (panels d, e, and f).

The data were analyzed in two ways. First, a series of generalized linear models for binomial distributions compared the proportion of *higher* vs. *same*, *higher* vs. *lower*, and *same* vs. *lower* responses for each condition. A second analysis assessed, for each condition, whether the response predicted under independence was more frequent than expected by chance. This second analysis was carried out in a series of linear models following a similar procedure to the coherence analysis above. To measure whether a response was more frequent than expected by chance under independence, we first coded whether a response conformed to the prediction under independence or not, and then subtracted this variable for observed conformance from the chance rate of conforming to the predicted response. With three response options, the chance rate was 1/3 on each trial. The data were analyzed using the glm and lmer functions for the R software environment (package lme4, Bates et al. (2015); R Core Team, 2017). Analyses were performed separately for each condition because the responses predicted under independence and under enhancement changed between conditions. The general rationale for model selection aimed to maximize the random structure justified by the design, as recommended by Barr et al. (2013). However, in this case it was only possible to include random intercepts for participants in the lmer models^1^.

The results show a complex picture that is not straightforward to group into findings concerning independence vs. enhancement, or explaining away vs. augmentation. We instead group the results into three domains that we think capture some of the most significant insights that can be gained from the findings, and which may explain some patterns of differences between experimental conditions.

### The Role of the Size of the Predicted Change

Consider first the conditions in which there was positive evidence for the effect (panels a, b, e, and f). Here the predictions under independence and enhancement coincide, so that any divergences from these predictions cannot easily be attributed to a violation of the assumption of causal independence.

Panels (a) and (e) show the results for when the effect (Ling fever) was certain to be present. Panels (b) and (f) show the results for when there was uncertain, indirect evidence that the effect was present because its consequence (rash) was present. Panels (e) and (f) further refer to the conditions in which one of the causes was absent. Responses in these latter two conditions showed a clear augmentation effect, in accordance with the predictions. That is, the dark bar in these panels tells us that the probability of a given cause was rated as higher upon learning that an alternative cause was absent.

Note that in these two cases the size of the predicted change under independence was larger than 10% (16.06% on average when the effect was present, and 10.95% on average when the consequence of the effect was present, as indicated through the horizontal scales at the bottom of the panels). Under enhancement, the size of the normative change would be expected to be even larger, but the extent to which it would be larger would depend on the strength of the causal interaction.

The augmentation effect was statistically significant. In panel (e) *higher* responses were more frequent than *same* responses (LR = 2.042, *z* = 2.865, *p* = 0.004, 95% CI [1.267,3.382]); and more frequent than *lower* responses (LR = 49, *z* = 3.853, *p* < 0.001, 95% CI [10.754,867.505]). The frequency of the predicted *higher* responses was above chance in this condition (*EMM* = 0.329, *F*(1,36) = 23.897, *p* < 0.001, *d* = 1.007, 95% CI [0.195,0.462]). In panel (f) *higher* responses were again more frequent than *same* responses (LR = 1.750, *z* = 2.187, *p* = 0.029, 95% CI [1.069,2.931]); and more frequent than *lower* responses (LR = 5.250, *z* = 4.299, *p* < 0.001, 95% CI [2.605,12.067]). The frequency of the predicted *higher* responses was also above chance in this condition (*EMM* = 0.234, *F*(1,36) = 11.105, *p* = 0.002, *d* = 0.690, 95% CI [0.095,0.374]).

The pattern of responses was less clear cut in panels (a) and (b). Here one of the causes is present and this “explains” the presence of the effect, leading to the prediction of a reduction in the probability of the other cause. But one can see that the size of the predicted change under independence is relatively small (3.86% on average when the effect was present, and 4.62% on average when the consequence of the effect was present, as shown in the horizontal scales at the bottom of the panels). Under enhancement, the normative size of the explaining away effect would be expected to be even smaller, albeit the extent of this decrement would again depend on the strength of the causal interaction. In line with this smaller normative change, fewer participants chose the normative *lower* response, and more participants chose the *same* response.

This pattern was corroborated statistically. For both panels (a) and (b), the frequency of the *lower* response did not differ significantly from that of *same* response (for (a): LR = 1.615, *z* = 1.992, *p* = 0.0546, 95% CI [0.997,2.666]). For (b): (LR = 1.429, *z* = 1.448, *p* = 0.148, 95% CI [0.885,2.337]); although the *lower* response was more frequent than the opposite *higher* response (For (a): LR = 0.231, *z* = −3.238, *p* = 0.001, 95% CI [0.086,0.524]. For (b): LR = 0.214, *z* = −3.424, *p* < 0.001, 95% CI [0.080,0.483]). The frequency of the *lower* response did not differ from chance in these two conditions (For (a): *EMM* = 0.018, *F*(1,36) = 0.067, *p* = 0.797, *d* = 0.051, 95% CI [−0.120,0.156]. For (b): *EMM* = 0.045, *F*(1,36) = 0.403, *p* = 0.529, 95%, *d* = 0.124, CI [−0.096,0.186]).

The pattern for panels (a) and (b) was similar to that for panel (h): the condition in which the consequence of the effect (rash) was absent and one of the causes was absent. Here the prediction under independence is that the opposite of augmentation occurs: the information that one of the causes is absent adds to the evidence for the absence of the effect, and the probability of the other cause decreases further. However, the size of the predicted change under independence was again relatively small (3.86% on average). In line with this, the *same* response was more frequent than the *lower* response (LR = 1.950, *z* = 2.428, *p* = 0.015, 95% CI [1.152,3.408]). The *lower* response was numerically more frequent than the opposite *higher* response, but this difference was not significant (LR = 0.750, *z* = −0.842, *p* = 0.400, 95% CI [0.377,1.458]). The frequency of the *lower* response was at chance level in this condition (*EMM* = −0.063, *F*(1,36) = 1.233, *p* = 0.274, *d* = 0.322, 95% CI [−0.176,0.050]).

The preceding results suggest people tended to respond in accordance with the probabilistic constraints given by the problem structure and in a way broadly consistent with the assumption of independence, but that differences in the frequency of relevant response options only reached significance when the normative size of the change was large enough to be noticeable (larger than 10% under independence). This was although participants made judgments only about the direction, and not about the size, of the change.

If this experiment had only tested the conditions in panels (a), (b), (e), and (f), involving positive evidence for the effect, then it would not have been possible to distinguish the role of the size of the normative change from whether this normative change was an increase (augmentation) or decrease (explaining away) of the probability of the cause asked for. That is, if we had only considered panels (a), (b), (e), and (f), then an alternative explanation for the difference in the pattern of results between (e) and (f) on the one hand, and (a) and (b) on the other, would have been that people find situations with negative evidence easier to think through than situations with positive evidence. But such an alternative explanation does not fit with the results for panel (h), which concern negative evidence and yet resemble the responses given to the cases of positive evidence in (a) and (b) more than those for negative evidence in (e) and (f). Considering the five panels together, a better, and simpler, explanation for the differences between conditions seems to be that they reflect differences in the size of the normative change. Further experiments varying the size of the normative change systematically across conditions would be necessary to further test this interpretation.

### The Probability of a Cause When the Effect Is Absent

Let us now turn to panels (c) and (g): the conditions in which the effect (Ling fever) was certain to be absent. Under the assumption of causal independence, the normative response in these two conditions is that there is no change. If the causes are instead positively correlated, then the normative response is explaining away for (c) and augmentation for (g). Finally, if the causes are interpreted as contributing independently to the effect but the absence of the effect is treated as uncertain (Oaksford and Chater, 2013; Over and Cruz, 2018), then the normative response is the opposite of explaining away and augmentation: an increase in the probability of a cause when another cause is present (c), and a decrease in the probability of a cause when another cause is absent (g). The conditions of panels (c) and (g) offer a unique opportunity for testing the contrasting predictions of the above three assumptions.

Consider first the condition in panel (c), where one of the causes is present. The *same* response predicted under independence was numerically more frequent than the *lower* response predicted under enhancement, but the difference was not significant (LR = 1.194, *z* = 0.727, *p* = 0.467, 95% CI [0.741,1.934]). The *same* and the *lower* response were both more frequent than the *higher* response predicted under the assumption of independence + uncertainty (*same* vs. *higher*: LR = 0.162, *z* = −4.133, *p* < 0.001, 95% CI [0.062,0.356]. *Lower* vs. *higher*: LR = 0.194, *z* = −3.682, *p* < 0.001, 95% CI [0.073,0.432]). The *same* response predicted under independence was above chance in this condition (*EMM* = 0.167, *F*(1,36) = 4.933, *p* = 0.033, *d* = 0.415, 95% CI [0.018,0.316]). Overall, the responses in this condition were in accordance with independence and, to a numerically lesser extent, with enhancement. In contrast, there was no evidence that participants followed the independence assumption while treating the information that the effect was absent as uncertain.

Turning to the condition in panel (g), where one of the causes is absent, the numerically most frequent response was again that there is no change, in line with the independence assumption. But the pattern was less clear cut, and no response option was significantly more frequent than the others (*same* vs. *lower*: LR = 1.333, *z* = 1.065, *p* = 0.287, 95% CI [0.788,2.286]. *Same* vs. *higher*: LR = 0.563, *z* = −1.953, *p* = 0.051, 95% CI [0.310,0.991]. *Lower* vs. *higher*: LR = 0.750, *z* = −0.923, *p* = 0.356, 95% CI [0.401,1.376]). The frequency of the *same* response was at chance level in this condition (*EMM* = 0.099, *F*(1,36) = 2.151, *p* = 0.151, 95%, *d* = 0.334, CI [−0.035,0.233]). Overall, participants seemed to have no clear common intuitions for the case in which both the effect and one of the causes was absent.

### Evidence Against Negative Evidence

Finally, consider the pattern in panel (d). Here there is uncertain evidence that the effect (Ling fever) is absent because its consequence (rash) is absent, and we then learn that one of the causes is present. For the parameters of the model, the predicted response under both independence and enhancement assumptions is that the opposite of explaining away occurs. This is because the presence of the cause undermines the uncertain evidence for the absence of the effect. The probability of the effect increases again, and with it also the probability of the other cause. The predicted size of the change under independence was 4.14% on average, which is not very large. Considering the findings from panels (a), (b), and (h), we can thus expect a relatively high frequency of same responses in this condition. The panel shows that although there was indeed a sizeable number of same responses, the most frequent response was instead the *lower* response, which is opposite to what had been predicted.

Statistically, the predicted *higher* responses were less frequent than the *same* responses (LR = 0.172, *z* = −3.630, *p* < 0.001, 95% CI [0.059,0.408]) and less frequent than the *lower* responses (LR = 0.125, *z* = −4.384, *p* < 0.001, 95% CI [0.043,0.288]). The frequency of the *higher* response was below chance in this condition (*EMM* = −0.266, *F*(1,36) = 59.504, *p* < 0.001, *d* = 1.728, 95% CI [−0.334,−0.197]).

The finding for this condition was surprising, and is the only one of the eight investigated in which responses seemed to deviate systematically from Bayesian predictions under both independence and enhancement assumptions. One possible explanation is that it constitutes a double negation effect. This effect, first described in research on deductive reasoning, refers to the finding that people make more errors drawing inferences when this requires negating a negation. That is, when it requires establishing that *not-not-A* = *A* (Evans and Handley, 1999; Oaksford et al., 2000). In a probabilistic extension of this idea, the present condition required participants to undermine negative evidence for an effect, and assess the consequences of this for the probability of a cause. However, the finding would have to be replicated and the conditions of its occurrence investigated further to determine the value of this explanation.

## Discussion

This study investigated people’s intercausal judgments in situations with several alternative causes for a common effect. We compared the predictions that follow from assuming that the causes contribute independently to the effect, with those that follow from assuming that the causes interact to some extent, enhancing each other’s contribution to the effect. In doing so, we took into account: (a) whether the information about the effect was considered certain or uncertain, (b) whether the evidence for the effect was positive or negative, and (c) whether one of the causes was present or absent. The resulting eight conditions were compared in a single experiment using a within participants design.

The experiment aimed to explore further people’s intuitions about explaining away and augmentation, and identify possible factors that could shed light on why previous studies have often found people’s responses to conform with the explaining away effects that follow from the independence assumption of the noisy-or, but to a lesser extent than predicted by normative models (Ali et al., 2011; Rehder, 2014; Rottman and Hastie, 2014).

Extant explanations for under-explaining away have pointed to possible differences in the interpretation of probability (Tešić et al., 2020), prior knowledge that changes the causal structure reasoned about, for instance by adding links between causes or additional intervening variables that must be active to allow an effect to occur (Mayrhofer et al., 2010; Rehder, 2014; Rottman and Hastie, 2014), and by positing that a subset of participants may represent the relations between variables as associative, and thus bidirectional, rather than as causal and unidirectional (Rehder and Waldmann, 2017). The latter has been referred to as the “rich get richer” principle because it implies that when one variable is present, this will increase the probability that variables connected to it will also be present, and vice-versa when a variable is absent (c.f. parallel constraint satisfaction networks, Glöckner et al., 2010).

The present results highlight two further possible reasons for the findings of under-explaining away, which we view as complementing rather than standing in competition to the explanations outlined above. The first is that people may not spontaneously interpret causes as contributing independently to the effect, as presupposed by the use of the noisy-or, but may sometimes instead interpret the causes as enhancing each other’s contribution, even in cases in which the materials are fictional and no explicit information suggesting any relation between causes is provided. On the one hand, this underlines the need to be careful when designing experiments, to make sure participants are really assuming causal independence before interpreting deviations from the predictions under independence as non-normative. On the other hand, it also points to the option of not trying to create materials for which independence unambiguously holds in the first place, and instead setting out to examine in more detail how people reason about causal structures with interacting causes.

The absence of a manipulation of the size of a correlation or interaction between causes was a limitation of the current study, and something worth pursuing in follow-up work. Such work could also include a dissociation of the two independence assumptions of the noisy-or separately, exploring separately people’s intuitions about (a) causes that covary, in the sense that the marginal probability that one cause is present changes as a function of the probability of another cause (Rottman and Hastie, 2014); and (b) causes that interact in their contribution to the effect, in the sense that whenever two causes happen to be present at the same time, the probability of the effect is increased or decreased to a greater extent than would be predicted by considering the impact of each cause independently (see also Fenton et al., 2019). An example of covariance would be a situation in which one cook is preparing soup, and the smell of the soup compels other cooks to enter the kitchen and start cooking more soup. An example of interaction would be a situation in which whenever soup happens to be cooked by more than one cook, the cooks start to work together; making the soup turn out better/worse than it would have been if they had been working independently.

Studies of reasoning about covarying and interacting causes are made more difficult by the lack of a single function from which to derive the CPT for the causal structure of interest. But this difficulty can be met by determining the size of each interaction effect, and then modifying an initial CPT based on independence to incorporate the interaction (Wellman and Henrion, 1993; Lemmer and Gossink, 2004; Fenton et al., 2019).

A second possible reason for the under-explaining away found in previous studies is that at least in some of these studies, the size of the normative change itself may have been too small to be subjectively relevant. Thus, people might be sensible to the probabilistic constraints posed by the structure of the problem, but our degrees of belief may be coarser than point probabilities, so that a larger change is necessary for it to be subjectively meaningful. The size of the normative change is not available in studies that do not include precise information about priors and causal power, and this information is of course often not available in real world situations (Rehder, 2014; Rottman and Hastie, 2014). However, the present findings suggest that in those cases in which the size of the normative change is not negligible, people’s responses do follow normative predictions in a consistent way.

**FIGURE 3:**
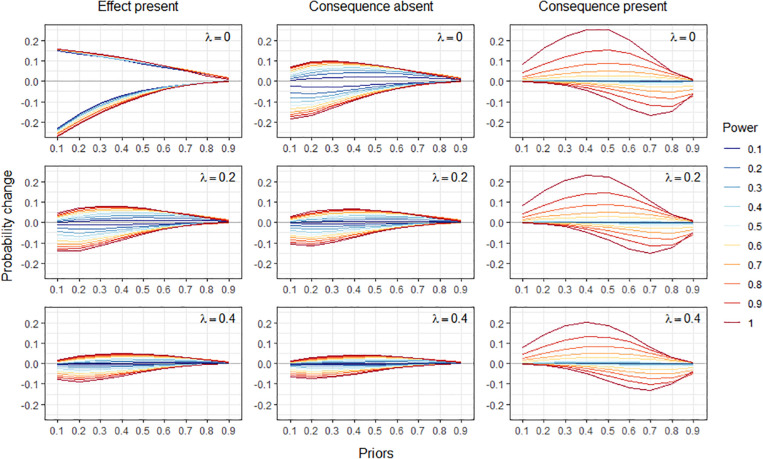
Normative changes in the probability of one cause when learning that another cause is present or absent, for a common-effect structure like that of Figure 1 but with equal priors and causal powers for each cause. Probability changes are shown as a function of the prior probabilities of the causes (x axis), causal power (separate lines), and the value of the leakage parameter (separate rows). Left column: explaining away and augmentation for the condition in which the effect is present. Middle column: opposite probability changes to those of explaining away and augmentation for the condition in which the consequence of the effect is absent. Right column: explaining away and augmentation for the condition in which the consequence of the effect is present. The condition in which the effect is absent is not shown here because probabilities remain invariant under the noisy-or in this case (c.f. Table 1).

As a further argument for the above interpretation, Figure 3 shows the size of the normative change that occurs under the assumption of independence for a causal structure like that of Figure 1. As in Figure 1, the leakage parameter for the consequence of the effect (rash) was set to 0.1, but unlike Figure 1, the causes were set to have equal priors and equal causal power for simplicity.

Figure 3 shows the size of the normative change for three of the four conditions of Table 1. The fourth condition: when the effect is absent, was not included in Figure 3 because it is associated with the prediction that the probabilities of the causes remain invariant, i.e., there is no normative change in this case.

The left column shows the condition in which the effect is known to be present – the most commonly studied case for explaining away in the literature. The middle column shows the condition in which the consequence of the effect is absent, and the right column shows the condition in which the consequent of the effect is present. For each condition, the size of the normative change is shown on the y axis as a function of the prior probabilities of the causes (on the x axis), the causal power of the causes (separate lines) and the leakage parameter λ (separate rows).

One can see that across the range of values these parameters can take, the size of the normative change only rarely reaches values higher than 20%, and it decreases as the value of the leakage parameter increases. When the effect is present or its consequence is absent, the size of the change tends to be larger for lower values of the prior, whereas this is not the case when the consequence of the effect is present. Overall, the size of the normative change increases with the power of the causes. When the effect is present, this effect of causal power is more or less evenly spread. In contrast, when the consequence is absent, it takes a causal power over 0.5 to obtain a non-negligible change at all, with higher values of causal power having increasing impact.

Note that as mentioned above, positive and negative normative changes arise under opposite conditions when the effect is present and when the consequence of the effect is absent. When the effect is present, negative changes correspond to the size of the explaining away effect, P(*cause B| effect & cause A*) - P(*cause B| effect*) < 0, and positive changes correspond to the size of the augmentation effect, P(*cause B| effect & not-cause A*) - P(*cause B| effect*) > 0. In contrast, when the consequence is absent, the normative probability changes go in the opposite direction: P(*cause B| effect & cause A*) - P(*cause B| effect*) > 0, and P(*cause B| effect & not-cause A*) - P(*cause B| effect*) < 0. This distinction is not visible in the graphs, which focus instead on illustrating the impact of causal power.

In the present study it was difficult to find model parameters for which the size of the predicted change was substantial across all experimental conditions in which a change was predicted, which included conditions in which the evidence for the effect was negative. But further studies could test this factor more explicitly by varying the size of the normative change within each condition and assessing the effect of this variation on participants’ probability judgments.

Future work could also assess the generalizability of the present findings by asking participants for numeric probability judgments under different information conditions in a dynamic reasoning setting, rather than for qualitative probability changes as was done here. This would also make it easier to build up the task more gradually for participants, for instance asking first about P(*effect*), then about P(*effect| not-cause A*), and finally about P(*effect| cause B & not-cause A*).

Finally, we found that responses were contrary to predictions under both independence and enhancement assumptions when a partial canceling of the effect of negative evidence was required. This unexpected finding resembles a probabilistic extension of the double negation effect from the deductive reasoning literature, and is worth investigating further. If replicated it may constitute a distinct source of error in probabilistic reasoning, beyond more frequently discussed sources such as those based on content effects, associative reasoning, and the use of heuristic task simplifications (Tversky and Kahneman, 1983; Oaksford, 2002; Glöckner et al., 2010; Rehder and Waldmann, 2017).

## Data Availability Statement

The datasets generated for this study are available on request to the corresponding author.

## Ethics Statement

The studies involving human participants were reviewed and approved by Ethics Committee of the Department of Psychological Sciences of Birkbeck, University of London. Written informed consent for participation was not required for this study in accordance with the national legislation and the institutional requirements.

## Author Contributions

NC implemented the study, analyzed the data, and wrote the manuscript. All authors contributed to informing the theoretical background and hypotheses and manuscript revision.

## Conflict of Interest

The authors declare that the research was conducted in the absence of any commercial or financial relationships that could be construed as a potential conflict of interest. The reviewer AT declared a shared affiliation with one of the authors DL to the handling editor at the time of review.

## Footnotes

1 For the binomial regression analyses, effect size estimates were provided through likelihood ratio values. For the linear model analyses, which in this case were intercept only models, effect sizes were estimated in the same way as for one-sample *t*-tests: as the ratio of the fixed effect of the intercept to its standard deviation.
